# Long noncoding RNAs in tumor stemness: emerging mechanisms and therapeutic opportunities

**DOI:** 10.3389/fgene.2026.1772938

**Published:** 2026-02-25

**Authors:** Eduardo Moraes Reis, Daniela Sanchez Bassères

**Affiliations:** Departamento de Bioquímica, Instituto de Química, Universidade de São Paulo, São Paulo, Brazil

**Keywords:** biomarkers, cancer stem cells, epigenetics, lncRNAs, RNA therapeutics, therapeutic targets, tumor heterogeneity, tumor plasticity

## Abstract

Cancer stem cells (CSCs) constitute a subpopulation of malignant cells with self-renewal and differentiation capabilities that drive tumorigenicity, metabolic adaptability, immune evasion, and therapeutic resistance, key factors contributing to metastasis and poor clinical outcomes. While genetic drivers of tumorigenesis are well-characterized, the epigenetic machinery sustaining the CSC state remains complex. Long noncoding RNAs (lncRNAs) represent a vast yet poorly understood class of regulatory molecules that influence gene expression at epigenetic, transcriptional, and post-transcriptional levels. Emerging evidence indicates that lncRNAs play a crucial role in shaping tumor cell plasticity and stemness-associated phenotypes. In this mini-review, we summarize current findings on how lncRNAs regulate CSC biology. We categorize their mechanisms of action, ranging from chromatin remodeling to the modulation of mRNA and protein stability. Furthermore, we discuss how the advent of high-resolution omics, including bulk tissue, single-cell, and spatial transcriptomics studies, is revolutionizing the identification CSC-associated lncRNAs and contributing to the development of clinically relevant biomarkers. Finally, we explore advanced methodologies for manipulating lncRNA expression, assessing the challenges and opportunities of lncRNA-directed therapeutics as a novel strategy to dismantle tumor plasticity and overcome drug resistance.

## 1 Introduction

Cancer progression and therapy resistance are inextricably linked to intratumor heterogeneity (ITH) and cancer cell plasticity (Beyes et al., 2021; Pan and Jia, 2021; Bhat et al., 2024). ITH is characterized by the presence of distinct subpopulations of cancer cells within a tumor, displaying a high degree of variation in cell states and phenotypes (Beyes et al., 2021; Bhat et al., 2024; Patel and Yanai, 2024). In contrast, cancer cell plasticity refers to the dynamic ability of cancer cells to reprogram their gene expression profiles, alter their behavior and identities, and adapt to microenvironmental cues (Pan and Jia, 2021; Bhat et al., 2024). Together, these processes represent a major obstacle to effective treatment, enabling subpopulations of cells to survive therapeutic pressures and allowing tumors to acquire metastatic and drug-resistant phenotypes (Beyes et al., 2021; Bhat et al., 2024).

**FIGURE 1:**
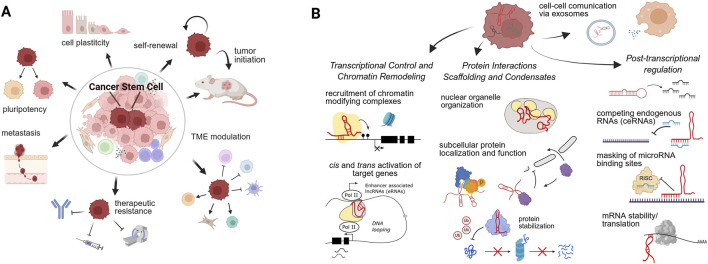
The multifaceted role of lncRNAs in Cancer Stem Cells (CSCs). **(A)** Hallmarks of the CSC phenotype. LncRNAs regulate core properties including self-renewal, pluripotency, phenotypic plasticity, and therapeutic resistance, ultimately driving tumor initiation and metastasis. **(B)** Molecular mechanisms of lncRNA action. The schematic illustrates three major modes of regulation (from left to right): Nuclear regulation, involving chromatin remodeling, enhancer activity, and transcriptional activation; Protein interplay, facilitating complex assembly, phase separation (condensates), and controlling protein stability/localization; and Post-transcriptional and extracellular regulation, where lncRNAs act as competing endogenous RNAs (ceRNAs), modulate mRNA stability, and mediate cell-cell communication via exosomes.

At the crossroads of these hallmarks lie cancer stem cells (CSCs), also referred to as tumor-initiating cells (Figure 1A). Characterized by their capacity for self-renewal and differentiation, CSCs are the primary engine of ITH (Evan et al., 2022; Yabo et al., 2022; Bhat et al., 2024). They generate heterogeneous tumor populations while retaining high phenotypic plasticity, allowing them to reversibly switch between stem-like and non-stem-like states (Kapoor-Narula and Lenka, 2022; Bhat et al., 2024). This bidirectional interconversion underpins the tumor’s ability to evade treatment, spread, and relapse (Kapoor-Narula and Lenka, 2022). Consequently, elucidating the molecular mechanisms sustaining CSC phenotypes is critical for developing durable therapies.

While genetic mutations drive tumor initiation, epigenetic regulation is paramount in controlling CSC transcriptional programs (Kapoor-Narula and Lenka, 2022; Bhat et al., 2024). Notably, long noncoding RNAs (lncRNAs), defined as transcripts longer than 200 nucleotides with limited or no protein-coding capacity, have emerged as key modulators of these epigenetic and transcriptional networks, fine-tuning gene expression patterns essential for stem-like features (Ciafrè et al., 2023; Yuan et al., 2025). They exert diverse functions at nearly every stage of gene regulation, from shaping the chromatin landscape to influencing protein stability and function (Ciafrè et al., 2023; Yuan et al., 2025). Consistent with their pleiotropic roles, lncRNAs have been implicated in multiple cancer hallmarks, including promoting CSC traits, metastasis, and therapy resistance (Ciafrè et al., 2023; Yuan et al., 2025). Their structural diversity and ability to interact with other RNAs or protein factors add layers of complexity to their study, but also provide opportunities for identifying novel mechanisms and therapeutic targets to counteract tumor plasticity.

Consequently, this mini-review aims to integrate the diverse molecular mechanisms of lncRNA action—from epigenetic remodeling to exosome-mediated communication—into a unified framework of cancer stem cell maintenance. Beyond describing individual pathways, we critically evaluate how these non-coding transcripts drive intratumoral heterogeneity and therapeutic resistance. Finally, we identify current knowledge gaps in lncRNA detection and discuss how emerging nucleic acid-based technologies, such as single-cell omics and CRISPR screening, are resolving these limitations to pave the way for clinical translation.

## 2 Literature selection criteria

To ensure a robust analysis of lncRNA-mediated stemness, we performed a targeted literature search on PubMed (up to December 2025) using combinations of the terms “Long noncoding RNA/lncRNA,” “Cancer Stem Cell,” and “Stemness.” This yielded an initial pool of approximately 280 non-review publications. To prioritize high-confidence drivers of stemness, we screened these studies for functional validation. Inclusion was strictly limited to studies that: (1) performed specific CSC-enrichment assays (e.g., tumorsphere formation, limiting dilution analysis, or flow cytometry-based sorting) following lncRNA gain- or loss-of-function; and (2) delineated a molecular mechanism of action. From this validated pool, we selected the representative examples discussed in Section 3 to illustrate the diversity of regulatory mechanisms, ranging from ceRNA networks to chromatin remodeling.

## 3 lncRNA-mediated mechanisms in CSC maintenance

To sustain the CSC phenotype, lncRNAs function as versatile regulatory hubs. They operate across distinct subcellular compartments, employing mechanisms that range from chromatin remodeling to the modulation of protein stability. A wide array of molecular mechanisms have been reported to explain how lncRNAs can regulate cancer stemness (Figure 1B), which will now be summarized.

### 3.1 Post-transcriptional regulation: the ceRNA network and mRNA stability

The most commonly reported mechanism for cancer stemness regulation involves lncRNAs functioning as competing endogenous RNAs (ceRNAs) for microRNAs (miRNAs), thereby de-repressing stemness-associated factors (Su et al., 2017; Peng et al., 2018; He et al., 2019; Liu et al., 2019; Tang et al., 2019; Guo et al., 2020; Wu et al., 2020; Zhan et al., 2020; He et al., 2021; Dong et al., 2023; Wang L. et al., 2023; Ci et al., 2024; Chu et al., 2025). For instance, in glioma stem-like cells (GSCs), SOX2OT sponges miR-194-5p and miR-122 to upregulate the oncogene SOX3, while LINC00115 acts as a miR-200 sponge to alleviate repression of ZEB1, promoting self-renewal and epithelial-mesenchymal transition (Su et al., 2017; Tang et al., 2019). However, a recurring challenge in validating these ceRNA networks is the issue of physiological stoichiometry; given that many lncRNAs are expressed at low copy numbers, it remains debated whether endogenous levels are sufficient to effectively sponge abundant miRNAs, or if these effects are merely artifacts of ectopic overexpression. Another issue is that ceRNA networks make the stemness role of lncRNAs hard to predict. Since miRNA and mRNA repertoires are tissue-specific, the same lncRNA can regulate distinct miRNA-mRNA networks depending on the cellular context. This plasticity likely explains why a single lncRNA may exhibit opposing stemness roles across different cancer types.

Beyond sponging, lncRNAs directly modulate mRNA stability (Guo et al., 2016; Ma et al., 2019; Miao et al., 2020; Wu et al., 2020; 2022; Liu et al., 2021; Zhu et al., 2021; Zhu et al., 2022; Pan et al., 2022; Shi et al., 2022; Zhang et al., 2022; Dong et al., 2023; Guan et al., 2025; He et al., 2025; Zhan et al., 2025). Lnc-ROPM, for example, binds the 3′-UTR of PLA2G16, enhancing its stability, thereby promoting breast cancer stem cell properties (Liu et al., 2021). Conversely, in pancreatic cancer, DDIT4-AS1 promotes the phosphorylation of UPF1 by preventing the binding of SMG5 and PP2A to UPF1, which decreases the stability of the DDIT4 mRNA and activates the mTOR pathway (Zhang et al., 2022). Finally, certain lncRNAs are packaged into exosomes to mediate cell-cell communication, propagating stemness phenotypes to neighboring cells within the tumor microenvironment (Li et al., 2019; Li Y. et al., 2022; Shi et al., 2023; Chen Y. et al., 2024; Yan et al., 2024; Zhan et al., 2025).

### 3.2 Protein interactions: scaffolding and condensates

LncRNAs often serve as molecular scaffolds, influencing protein stability, protein activity and the formation of subcellular structures. Several lncRNAs inhibit proteasome-mediated degradation of key oncoproteins in CSCs, either by directly regulating ubiquitination or modulating other post-translational modifications required for ubiquitination (Zhu P. et al., 2016; Luo et al., 2018; Li Y.-P. et al., 2022; Tsang et al., 2022; Wei et al., 2022; Wang L. et al., 2023; Huang et al., 2024). A notable example of protein activity modulation is UCA1, which scaffolds the interaction between hnRNPA2B1 and KRAS in pancreatic cancer, enhancing KRAS phosphorylation and stem cell maintenance (Liu et al., 2019). Furthermore, recent studies highlight the role of lncRNAs in phase separation. NEAT1 induces nuclear paraspeckle formation required for CSC clonogenicity and self-renewal (Bhattacharya et al., 2024) and binds the intrinsically disordered region of YAP to promote liquid-liquid phase separation biomolecular condensates, driving tumorigenesis (Chen et al., 2025). Nonetheless, while these interactions are robustly characterized in *vitro* models, the stability of these lncRNA-protein complexes within the hypoxic and metabolically stressed tumor microenvironment remains less understood, highlighting a need for more patient-derived xenograft (PDX) validation.

### 3.3 Nuclear mechanisms: transcriptional control and chromatin remodeling

In the nucleus, lncRNAs act as potent epigenetic modulators. They may recruit transcription factors directly to promoters—such as HOTAIR recruiting AR, or LINC00261 recruiting GATA6 — to activate stemness genes like GLI2 and SOX2 (Bai et al., 2021; Chen et al., 2022). They may also recruit RNA polymerase directly to promoters (Jiang et al., 2020; Li et al., 2023).

More profoundly, lncRNAs guide chromatin remodeling complexes to specific loci (Wang et al., 2015; Wang et al.,2016; Wang et al.,2019; Wang et al.,2021; Wang et al.,2024; Chen et al., 2019; Chen W. et al., 2024). LncTCF7 recruits the SWI/SNF complex to the TCF7 promoter to increase chromatin accessibility (Wang et al., 2015), while HAND2-AS1 recruits the INO80 complex to activate BMP signaling in liver CSCs (Wang et al., 2019). They also modulate histone methylation. HOTAIR recruits EZH2 (PRC2 complex) to repress differentiation genes via H3K27 trimethylation (Wang et al., 2021; Wang et al.,2024), whereas HotairM1 blocks PRC2 binding at the HOXA1 promoter to maintain expression (Li et al., 2020). Beyond histone methylation, lncRNAs can also regulate histone acetylation and DNA methylation. Lnc34a recruits Dnmt3a via PHB2 and HDAC1 to methylate and deacetylate the miR-34a promoter, epigenetically silencing miR-34a expression (Wang et al., 2016) and MIR31HG recruits the WDR5/MLL3/P300 complex in lung cancer stem cells to activate Gli2 expression by histone H3K4 methylation and H3K27 acetylation (Chen W. et al., 2024). Additionally, 3D chromatin architecture is influenced by lncRNAs. CUDR promotes β-catenin expression by forming β-catenin promoter-enhancer DNA looping mediated by the CUDR-CTCF complex (Gui et al., 2015) and CASCADES traps YY1 at the SOX2 promoter to form a chromatin loop that sustains glioblastoma stem cell identity (Shahzad et al., 2025).

The diversity of these epigenetic mechanisms underscores a major complexity in the field: the function of a single lncRNA is often dictated by its specific splicing isoforms or subcellular localization (Statello et al., 2021), which can vary significantly between CSC subpopulations even within the same tumor (Zhang et al., 2020; Liu et al., 2021).

### 3.4 Emerging mechanisms: splicing, miRNA processing and peptide action

Recent evidence implicates that lncRNAs regulate cancer stemness by regulating alternative splicing. RAB30-DT stabilizes the splicing kinase SRPK1, driving splicing reprogramming (Si et al., 2025), while the long isoform of LHFPL3-AS1 interacts with PTBP1 to promote its own splicing, creating a feed-forward loop for stemness (Zhang et al., 2020).

LncRNAs also regulate stemness by modulating miRNA biogenesis. ADAMTS9-AS2 blocks LIN28B from processing pri-let-7, allowing mature let-7 to suppress the oncogene MYCN (Liu et al., 2023) and HULC increases the binding of the RNA methyltransferase METTL3 to pri-miR675, enhancing the expression and maturity of miR675 (Wang et al., 2020). Finally, MIAT interacts with Mtdh to regulate the biogenesis/abundance of microRNAs involved in cancer initiation (Peng et al., 2022).

LncRNAs can code for small biologically active peptides to promote stemness. LINC00511 codes for a 133 amino-acid peptide that activates the Wnt/β-catenin pathway to promote breast cancer invasion and stemness (Tan et al., 2023).

These findings underscore the complexity of lncRNA-mediated regulation in cancer stemness and highlight the importance of future mechanistic studies. Notably, some transcripts, such as *MIR22HG*, *SOX2OT* and LINC01106 exhibit dual roles, simultaneously acting as ceRNAs and protein scaffolds (Guo et al., 2020; Dong et al., 2023; Ci et al., 2024). A network-based understanding of lncRNA-based mechanisms in cancer stemness is essential for uncovering CSC-specific vulnerabilities. As we discuss in the following section, advancing our detection capabilities via omics technologies is the next step toward translating this knowledge into clinical biomarkers.

## 4 Technological advances for identifying CSC-Associated lncRNAs

**FIGURE 2:**
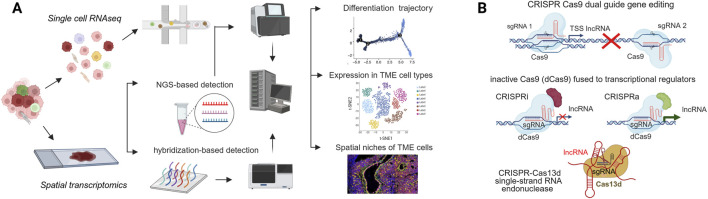
Advanced methods for identitifcation and functional characterization of stemness associated lncRNAs. **(A)** Advanced transcriptomic profiling. Single-cell RNA sequencing (scRNA-seq) enables the resolution of cellular heterogeneity and differentiation trajectories, while spatial transcriptomics maps CSCs and lncRNA expression within the tumor microenvironment (TME) niche. **(B)** Functional manipulation strategies. CRISPR-based technologies used to validate lncRNA function, including genomic deletion (dual-guide Cas9), transcriptional repression/activation (CRISPRi/a), and direct RNA targeting (Cas13d).

The development of single cell (scRNAseq) and spatial transcriptomics (ST) technologies paved the way for the analysis of the composition and functional states of the individual cellular components of the tumor microenvironment (TME). Current ST approaches can be broadly divided into image-based methods, such as *in situ* hybridization (ISH) and *in situ* sequencing (ISS), which directly visualize RNA transcripts, and barcode-based methods, which capture transcripts using spatially encoded oligonucleotide barcodes to retain positional information (Figure 2A). These complementary strategies provide unprecedented resolution for investigating tissue architecture and tumor heterogeneity and hold great promise to uncover molecular vulnerabilities in CSC populations (Huang et al., 2025). These methods have provided further evidence of inter and intratumoral heterogeneity in cell composition and activation states (Pei et al., 2025). Analysis of cell-cell communication provided novel insights regarding stromal-immune interactions (Ho et al., 2019; Dang et al., 2023), whereas spatial mapping of expressed genes revealed the existence of cell niches driving tumor progression and therapy resistance (Ren et al., 2023). Trajectory analysis (pseudotime) of scRNA-seq data from tumor and non-malignant tissue allows the identification of subpopulations of cells in different states of differentiation and of stemness-associated gene sets (Jiang et al., 2021; Ren et al., 2021). The data generated in these experiments is a valuable resource to validate, in the context of the TME, previously identified stemness-associated biomarkers, including both protein coding genes and lncRNAs, as well as to optimize computational tools for deconvolution analysis of tumor bulk RNAseq (Malta et al., 2018; Tirosh and Suva, 2024; Deng et al., 2025; Liu et al., 2025; Shi et al., 2025).

Single cell and spatial resolution technologies, coupled with integrative bioinformatics pipelines, can facilitate the generation of catalogues of lncRNAs expressed in each TME cell subtype, offering insights into conserved versus tumor-specific stemness-associated lncRNA functions. As an example, a signature of 111 cell-specific lncRNAs reflecting tumor, immune, and stromal contributions for pancreatic adenocarcinoma was identified, many of which were associated with patient outcomes and validated across multiple datasets (Dang et al., 2023). Several of these lncRNAs were associated with epithelial-mesenchymal transition (EMT), metabolism, and immune signaling, suggesting potential links to stemness-related pathways. The observed intratumoral heterogeneity and cell subclusters may also reflect stem-like populations within PDAC, where lncRNAs could act as regulators of stemness, therapy resistance, and tumor progression. In another study, single cell analysis from triple-negative breast cancer (TNBC) patients with either tumor elimination or persistence after neoadjuvant chemotherapy (NAC) identified hundreds of lncRNAs deregulated in persistent cases, including MALAT1 transcripts. Functional experiments showed that CRISPR/Cas9-mediated MALAT1 promoter deletion in TNBC cells increased sensitivity to paclitaxel and doxorubicin, implicating MALAT1 in chemoresistance (Shaath et al., 2021). The potential of ST to identify clinically relevant lncRNAs was demonstrated by the identification of three lncRNAs (LINC01978, PLAC4, and LINC01303) highly elevated in metastatic tissues, which were cross-validated in bulk and single-cell RNAseq and independent ST datasets, and may serve as prognostic biomarkers for metastatic progression (Pinkney et al., 2024). However, a major limitation of these observational atlases is that spatial co-localization implies, but does not prove, functional interaction. High-throughput perturbation mapping (e.g., Perturb-seq, Dixit et al., 2016) will be required to distinguish true stemness drivers from bystander lncRNAs that are merely upregulated during dedifferentiation. Another challenge in studying lncRNAs is that the lower expression level and more tissue-restricted pattern of lncRNAs compared to protein coding genes, coupled to the enrichment of polyadenylated RNAs during NGS library preparation, results in lncRNAs being underrepresented in the scRNAseq and ST data sets. Notwithstanding, the availability of increasing amounts of scRNAseq datasets has led to the development of resources to facilitate the exploration of lncRNAs within the TME. LnCeCell 2.0 (http://bio-bigdata.hrbmu.edu.cn/LnCeCell) provides a comprehensive database of lncRNA-associated ceRNA networks across different tumor types and normal tissues, whereas PDACLncDB (https://www.maherlab.com/pdaclncdb-overview) is a curated database for exploration of lncRNA landscapes in pancreatic ductal adenocarcinoma, highlighting intratumoral heterogeneity and clinically relevant lncRNAs.

## 5 Therapeutic potential of targeting lncRNAs in CSCs

In contrast to protein-coding genes, lncRNAs display greater specificity in their expression across cell types and biological contexts, which makes them promising candidate biomarkers of tumor behavior and potential adjuvant targets in cancer therapy. In the context of tumor stemness, lncRNAs play critical roles in regulating the self-renewal, differentiation, and plasticity of CSCs, thereby sustaining the CSC phenotype and contributing to tumor heterogeneity, progression, and resistance to therapy.

The characterization of lncRNA function in cell lines has extensively relied on silencing mediated by antisense oligonucleotides (ASO) or small interfering RNAs (siRNAs). These strategies have shown promise in silencing oncogenic lncRNAs such as HOTAIR and MALAT1, leading to reduced CSC viability and tumorigenicity in preclinical models (Jiao et al., 2015; Bai et al., 2021). Preclinical and clinical evidence supporting the therapeutic targeting of lncRNAs in CSCs is steadily accumulating. In breast cancer models, silencing HOTAIR has been shown to impair CSC self-renewal and reduce metastatic potential (Pádua Alves et al., 2013). In pancreatic cancer, MALAT1 expression increases the CSC population, maintains self-renewal capacity, decreases the chemosensitivity to anticancer drugs, and accelerates tumor angiogenesis *in vitro* (Jiao et al., 2015). In colorectal cancer, H19 promotes tumor stemness and chemoresistance by activating the β-catenin pathway, acting as a ceRNA for miR-141. Interestingly, the H19 is exported from cancer-associated fibroblasts (CAFs) in exosomes that are internalized by tumor cells (Ren et al., 2018). In hepatocellular carcinoma, inhibition of the lncRNA DANCR suppresses stemness markers and reduces tumorigenicity *in vivo* (Yuan et al., 2016). These findings underscore the functional importance of lncRNAs in sustaining CSC phenotypes and validate them as actionable targets.

The advent of clustered regularly interspaced short palindromic repeats (CRISPR)–based genome editing tools has transformed the ability to interrogate the function of lncRNA loci (Rinn and Chang, 2020) (Figure 2B). The use of conventional single-guide CRISPR-Cas9 has limitations for lncRNA targeting, since small indels may not disrupt lncRNA function, whereas extensive deletions may eliminate DNA regulatory elements present in the lncRNA locus. As an alternative, dual sgRNA guide strategies to delete the promoter of lncRNAs (Aparicio-Prat et al., 2015; Zhu S. et al., 2016), or target splicing sites (Liu et al., 2018) have been devised and adapted for pooled library functional screening of lncRNAs.

Critically, discrepancies often arise between RNAi-mediated knockdown and traditional CRISPR-Cas9 knockout studies. Unlike acute silencing, permanent genomic deletion can trigger genetic compensation mechanisms (El-Brolosy et al., 2019) that mask lncRNA phenotypes. This limitation highlights the superiority of alternative strategies that minimize compensatory responses by modulating expression without altering the DNA sequence, such as RNA-targeting CRISPR-Cas13 systems (Abudayyeh et al., 2017) or, more prominently, epigenetic silencing tools. Accordingly, modified CRISPR-Cas9 systems based on an inactive Cas9 fused to transcriptional activators (CRISPRa) or silencers (CRISPRi) have emerged as powerful tools to activate or disrupt lncRNA loci epigenetically, offering a more durable and potentially reversible therapeutic option compared to ASO/siRNA and CRISPR-Cas9 deletions, respectively. A CRISPRi screen in glioblastoma identified the lncRNA LINC03045 as a regulator of invasion and stemness-related pathways (Tsung et al., 2024). Similarly, an *in vivo* CRISPRi screen in cutaneous squamous cell carcinoma (cSCC) uncovered a portfolio of lncRNAs essential for tumor growth and progression, highlighting their role in maintaining stem-like properties (Kim et al., 2024). Likewise, a genome-wide CRISPRa screen identified lncRNAs whose activation promoted stem-like phenotypes and therapy resistance (Bester et al., 2018). More recently, Wang et al. performed a CRISPRa gain-of-function screen of 9,744 lncRNAs in melanoma cells co-cultured with CD8^+^ T cells, uncovering lncRNAs that regulate tumor immune evasion and stemness-related survival pathways (Wang et al., 2022). It is likely that CRISPR-based tools will increasingly serve as indispensable approaches to confirm the functional roles of long noncoding RNAs in stemness-associated phenotypes, enabling precise validation of their contributions to pluripotency, differentiation, and regenerative potential.

Clinically, the field of lncRNA-based therapies is still incipient. Functionalized nanoparticle-based systems able to specifically and efficiently deliver drugs to target CSC populations will be essential for the development of lncRNA-targeted therapeutics. In this regard, the development of lipid nanoparticles mimicking exosome membranes is an emerging strategy, leveraging the natural biocompatibility and targeting capabilities of tumor-derived extracellular vesicles (Munagala et al., 2021). Despite these advances, the delivery problem remains the primary bottleneck; the dense extracellular matrix and high interstitial pressure of solid tumors severely limit the penetration of lipid nanoparticles (Jain and Stylianopoulos, 2010; Mitchell et al., 2021), often restricting delivery to the perivascular niche rather than the deep hypoxic core where quiescent CSCs reside.

Consequently, rather than relying on lncRNA inhibitors as monotherapies, the most pragmatic clinical strategy is to exploit potential synergies with existing therapies. Integrating lncRNA-targeted approaches into broader regimens can sensitize otherwise recalcitrant populations. CSCs are notoriously resistant to conventional therapies, contributing to relapse and metastasis. Targeting lncRNAs that mediate resistance mechanisms can sensitize CSCs to chemotherapy and radiotherapy. Immunotherapy could also benefit from lncRNA modulation, as certain lncRNAs regulate the expression of immune checkpoint proteins, thereby influencing the immunosuppressive tumor microenvironment. By disrupting these pathways, lncRNA-targeted therapies may enhance immune recognition and clearance of CSCs. Furthermore, combining lncRNA inhibition with epigenetic drugs could synergistically dismantle the transcriptional networks that sustain CSC identity. These combinatorial strategies hold promise for overcoming therapeutic resistance and achieving more durable responses in cancer treatment (Wang N. et al., 2023).

## 6 Discussion

The growing recognition of lncRNAs as important regulators of tumor stemness has opened new conceptual and therapeutic frontiers, yet several challenges remain. One of the most striking insights is the sheer mechanistic diversity of lncRNAs—ranging from chromatin remodeling and transcriptional scaffolding to post-transcriptional mechanisms of gene expression control, interacting with DNA, RNAs or proteins. A critical unresolved question is how to define universal targeting strategies given this versatility; the fact that the same lncRNA may exert opposing effects depending on cellular context raises fundamental doubts regarding the feasibility of broad-spectrum interventions. Future efforts must determine whether integration of high-resolution transcriptome profiling with other omics datasets—such as epigenomic, proteomic, and metabolomic data—using state-of-the-art artificial intelligence (AI) and machine learning algorithms can successfully identify context-specific targets and predictors of therapy response (You et al., 2025).

Significant gaps also persist regarding the functional annotation of lncRNAs. Despite the explosion of transcriptomic data, especially from single-cell and spatial platforms, the underrepresentation of lncRNAs due to technical biases in sequencing protocols continues to limit discovery. Moreover, distinguishing functional lncRNAs from transcriptional noise remains a methodological hurdle, particularly in high-throughput CRISPR screens where genomic deletions may inadvertently disrupt regulatory DNA elements rather than the RNA product itself.

Therapeutically, while antisense oligonucleotides and CRISPR-based tools have demonstrated proof-of-concept efficacy in preclinical models, their translation to the clinic is still in early stages. The specificity of lncRNA expression offers a theoretical advantage for minimizing off-target effects, yet a key translational bottleneck is the lack of robust delivery systems, especially for targeting rare and spatially restricted CSC populations. While lipid nanoparticles and exosome-mimetic carriers show promise, critical questions remain regarding their ability to penetrate the dense tumor matrix and their clinical validation is pending (Tenchov et al., 2022).

A compelling yet underexplored opportunity lies in combining lncRNA-targeted approaches with existing therapies. The potential to sensitize CSCs to chemotherapy, radiotherapy, or immunotherapy by dismantling lncRNA-mediated resistance circuits is conceptually attractive. However, it remains unclear whether targeting a single lncRNA is sufficient given the redundancy and adaptability of CSC regulatory networks. Addressing this uncertainty requires a deeper understanding of lncRNA cooperativity within stemness programs to identify the most potent combinatorial strategies.

In sum, lncRNAs represent both a challenge and an opportunity: their complexity mirrors the plasticity of CSCs but also provides a rich substrate for therapeutic innovation. To resolve the critical knowledge gaps impeding this translation, future research must prioritize functional validation, context-specific targeting, and integrative approaches that account for the dynamic nature of tumor ecosystems.
